# 2272: Pilot study: Implementing Brief Dialectical Behavior Therapy (DBT-A) group skills training in a public and alternative high school setting

**DOI:** 10.1017/cts.2017.124

**Published:** 2018-05-10

**Authors:** Tamika Zapolski, Matthew C. Aalsma, Michelle Salyers, Dennis Watson

## Abstract

OBJECTIVES/SPECIFIC AIMS: Engagement in risky behaviors is not uncommon among adolescents. Two factors associated with risk taking are difficulty regulating emotions and impulsivity. Moreover, youth who exhibit higher scores on impulsivity-like personality traits (ie, negative urgency, positive urgency, sensation seeking, lack of premeditation, and lack of perseverance) are at even heightened risk. An effective intervention decreasing risk-taking behavior among adolescent populations in clinical settings is Dialectical Behavioral Therapy for Adolescents (DBT-A), which teaches skills on emotion regulation, distress tolerance, and mindfulness. However, DBT-A has yet to be tested as an intervention for youth in a nonclinical setting. The current study aimed to fill this gap in the literature. METHODS/STUDY POPULATION: A 9-week DBT-A skills group was implemented in a public high school classroom (7th-8th graders; N=41) and an alternative high school for at risk youth (7th-12th graders; n=21). Of the 41 youth from the public high school classroom participated, with preintervention and postintervention data provided by 30 participants (retention of 73%). RESULTS/ANTICIPATED RESULTS: Results found a significant increase in mindfulness skills and marginally significant increase in emotion regulation skills. Although there was not an overall change in risky behavior among participants, those who were higher on lack of premeditation and positive urgency showed steeper improvements on the skills. The second study at the alternative high school is currently underway, with no current results to report. DISCUSSION/SIGNIFICANCE OF IMPACT: This study will demonstrate that DBT-A skills training is feasible in a school-based setting and shows promising preliminary evidence of decreasing risk of engagement in risky health behaviors among adolescents, particularly among high-risk youth.

